# Credit Card Fraud Detection: An Improved Strategy for High Recall Using KNN, LDA, and Linear Regression

**DOI:** 10.3390/s23187788

**Published:** 2023-09-10

**Authors:** Jiwon Chung, Kyungho Lee

**Affiliations:** School of Cybersecurity, Korea University, Seoul 02841, Republic of Korea; 0000againism@korea.ac.kr

**Keywords:** recall analysis, sensitivity analysis, true positive rate analysis, credit card fraud detection, KNN, LDA, linear regression

## Abstract

Efficiently and accurately identifying fraudulent credit card transactions has emerged as a significant global concern along with the growth of electronic commerce and the proliferation of Internet of Things (IoT) devices. In this regard, this paper proposes an improved algorithm for highly sensitive credit card fraud detection. Our approach leverages three machine learning models: K-nearest neighbor, linear discriminant analysis, and linear regression. Subsequently, we apply additional conditional statements, such as “IF” and “THEN”, and operators, such as “>“ and “<“, to the results. The features extracted using this proposed strategy achieved a recall of 1.0000, 0.9701, 1.0000, and 0.9362 across the four tested fraud datasets. Consequently, this methodology outperforms other approaches employing single machine learning models in terms of recall.

## 1. Introduction

Fraud involves criminal deception and the use of false representation to unjustly gain an advantage or harm the rights and interests of others [1]. The proliferation of online transaction methods and technologies has led to a surge in international online fraud, resulting in substantial financial losses. The accessibility of online transaction systems and Internet of Things (IoT) devices has driven up transaction volumes, consequently escalating the risk of fraud [2]. For instance, credit card fraud cases have been on the rise in the US [3].

According to the 2023 Credit Card Fraud Report, the percentage of US credit and credit card holders who had fallen victim to fraud at some point in their lives increased to 65% in 2022, up from the 58% reported in 2021 [4]. Credit card fraud is not limited to the US; it is a global issue, including in the Republic of Korea [5].

Given the prevalence of fraud, there is a pressing need for robust fraud detection systems. Broadly, fraud detection falls into two categories: misuse and anomaly detection [6]. Misuse detection employs machine-learning-based classification models to differentiate between fraudulent and legitimate transactions. Conversely, anomaly detection establishes a baseline from sequential records to define the attributes of a typical transaction and create a distinctive profile for it. This paper presents a strategy for misuse detection utilizing a blend of K-nearest neighbor (KNN), linear discriminant analysis (LDA), and linear regression (LR) models.

**Figure 1:**
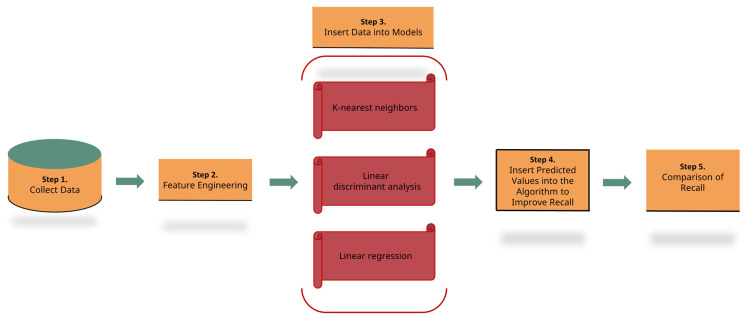
Proposed credit card fraud detection model.

The contributions of this study are as follows:

- We conducted experiments employing three machine learning algorithms (KNN, LDA, and LR) as well as our integrated algorithm, attaining superior recall in detection performance. Thus, this methodology could be adopted in other fields where recall is crucial. It is depicted abstractly in Figure 1.
- We applied the proposed approach to four extensive datasets concerning credit card fraud, including a real-world dataset.
- We verified that our methodology outperforms individual machine learning models in terms of recall using PyCaret, an automated machine learning library.

The remainder of this paper is structured as follows: Previous studies concerning the application of KNN, LDA, and credit card fraud detection are outlined in Section 2. Section 3 is divided into two parts: Section 3.1 details the characteristics and processing of the four datasets. In Section 3.2, the KNN, LDA, and LR models are explained, along with our supplementary algorithm developed for enhanced recall, presented in pseudocode. The combined methodology is also detailed. The results are summarized in Section 4, with a specific focus on comparing our method with individual machine learning models for the four datasets in terms of recall and accuracy. Section 5 outlines the limitations of our study, and we conclude our investigation in Section 6.

## 2. Related Studies

### 2.1. Importance of Recall

Recall, also referred to as sensitivity and the true positive rate (TPR), holds significant importance in fraud detection. Ensuring accurate fraud detection is vital, especially in preventing the misclassification of genuine fraud cases as non-fraud instances. Recall is computed using Equation (1).

(1) $$\mathrm{Recall}=\frac{TP}{TP+FN}$$

Lei and Ghorbani [7] introduced ICLN, an unsupervised clustering algorithm, and SICLN, a supervised clustering algorithm, for fraud detection. They selected these models based on their emphasis on recall, as in fraud detection, recall holds greater importance than overall accuracy and precision. Prasetiyo et al. [8] also conducted experiments in fraud detection, with recall as the evaluation metric, aligning with the dataset’s characteristics. They achieved a recall of 84.52% and an F1 score of 84.93%. Gupta et al. [9] focused on cervical cancer detection, where recall is crucial due to the potentially fatal consequences of misclassification. Therefore, they adopted recall as the primary measure to assess their model’s effectiveness.

Building on this emphasis on recall, it is worth discussing its economic implications, particularly in the realm of financial institutions. Even a few instances of fraud can inflict substantial financial losses on financial institutions and erode customer trust. The success of a single credit card fraud event can precipitate a cascade of subsequent fraudulent activities. Therefore, accurately detecting each case of fraud is paramount. By achieving a high recall value and minimizing false negatives, economic benefits and cost-saving measures can be reaped.

### 2.2. Classification Using KNN and LDA

We chose to employ the KNN and LDA due to their proven usefulness in terms of accuracy and recall, as indicated by previous literature. These models have demonstrated excellent performance across various applications [10,11,12]. For instance, Murugappan [10] utilized KNN and LDA for electromyogram (EMG) signal analysis to identify human emotions considering their simplicity, achieving maximum classification rates of 90.83%, 100%, 94.17%, and 90.28% for the emotions of disgust, happiness, fear, and neutrality, respectively. Starzacher and Rinner [11] applied KNN and LDA in the context of classifying vehicles for traffic monitoring, demonstrating promising results with a low false positive rate. Their study highlighted that KNN and LDA can yield high recall. Similarly, Lopez-Bernal et al. [12] opted for KNN and LDA because of their relative simplicity compared to more advanced machine learning algorithms. They utilized these models with datasets related to heart disease, banknote authentication, and cancer, achieving a maximum recall of 1.000 with KNN and 0.9999 with LDA. Taken together, these results suggest that KNN and LDA are well-suited for use in credit card fraud detection. While tree-based machine learning models such as decision tree (DT), random forest (RF), and gradient boosting classifier (GBC) have been widely used for credit card fraud detection [13,14,15,16,17], KNN and LDA, although less commonly used in this domain, offer simplicity and strong performance in terms of recall. Therefore, we decided to combine these models with LR. The inclusion of LR was driven by our discovery that it could enhance recall in a specific manner. Further details about it are provided at the end of Section 2.3.

### 2.3. Shortfalls of Previous Studies

While methodologies aiming to achieve high performance using machine learning models have been extensively studied, research on credit card fraud detection has predominantly focused on a single dataset, often prioritizing accuracy and AUC score as primary performance indicators. However, recall cannot be directly deduced from the AUC score. As a result, recall has garnered relatively limited attention in the context of credit card fraud detection. To address this gap, this paper introduces a methodology explicitly designed to achieve high recall in detecting credit card fraud.

In our experiments, we applied our approach to the datasets [18,19,20,21] used in some of the studies we reviewed. This choice was made to facilitate performance comparison and assess the potential for generalization. The studies we reviewed are limited to those employing unique methodologies, such as voting-like methods frameworks. Furthermore, we focused on recent publications within the last two years and endeavored to select those that utilized publicly available datasets. It is worth noting that many of the publicly available datasets are synthetic due to the scarcity of commercial data regarding security and privacy concerns [22]. As a result, many studies resort to synthetic data. Table 1 provides a summary of the studies we examined.

Zahoora et al. [23] successfully deployed a sophisticated heterogeneous voting ensemble named DCAE-ZSL-HVE, leveraging the capabilities of Contractive Autoencoder (CAE) for the detection of zero-day ransomware attacks. They achieved an impressively high recall value of approximately 0.95. Their approach notably outperformed traditional machine learning techniques in comparative evaluations. Attribute Learning (AL) and the Inference Stage (IA) were integral components of their methodology, systematically enhancing the recall metrics. However, given the limited scope of the dataset they employed, it remains uncertain whether their method would generalize effectively to vastly different datasets.

Verma and Chandra [24] proposed a RepuTE Framework tailored to bolster trust in the fog computing layer near users. This framework deploys a soft-voting ensemble learning model to classify and predict DoS/DDoS and Sybil attacks. The framework exhibited remarkable performance in test results, surpassing existing methods with a 99.99% accuracy rate. This outcome underscores its potential for reputation-based attack filtration in the IoT domain. However, its applicability in scenarios marked by a severe class imbalance, such as credit card fraud datasets characterized by a highly skewed ratio of fraudulent to legitimate transactions, remains uncertain.

Malik et al. [25] employed a voting-like technique with the dataset [21]. They focused on attaining optimal ROC values using amalgamating models, including LR, RF, DT, XGBoost, naive Bayes (NB), support vector machine (SVM), and light gradient boosting machine (LGBM), with the AdaBoost model. However, an area of concern arises from their omission of accurate details. As elaborated later in this study, while the NB model demonstrates commendable ROC and recall values with this dataset, its accuracy conspicuously remains low.

Jiang et al. [26] introduced a novel unsupervised attentional anomaly detection network-based framework for credit card fraud detection. Their model combines a generator and a discriminator: the former incorporates an autoencoder, while the latter contributes to an adversarial training setup. They contend that their model excels in generalization compared to other frameworks, a claim substantiated by its impressive precision and AUC scores. Nonetheless, although the model achieves a recall value of approximately 0.75, there is room for enhancing this particular metric.

Akshaya et al. [27] conducted a comparative performance analysis of various models, including logistic regression, GBC, KNN, RF, and voting classifier, for credit card fraud detection. In their study, the voting classifier emerges as superior in terms of accuracy and F1 score when compared to the aforementioned models. However, there is potential for improvement in the voting classifier’s recall performance, indicating an avenue for refinement.

Cai and He [28] showcased notable results by integrating Google’s TabNet, a deep neural network, with XGBoost for credit card fraud detection. They partitioned the dataset [16] based on the columns “transaction” and “identity”, modifying the “fraud” field to “fraud” if the transaction was identified as such. This hybrid methodology outperformed standalone applications of either TabNet or XGBoost in terms of accuracy and AUC score. Yet, there was no explicit mention of the recall value, and the pronounced emphasis on accuracy somewhat limited the analysis.

Nguyen et al. [29] devised an advanced framework for real-time credit card fraud detection. Prior to feeding data into the deep learning model, they implemented a distinction mechanism to classify credit card users as either longstanding or newcomers. Their meticulous approach extended to data preprocessing, including dimensionality reduction and data normalization. However, the use of deep learning models came with a considerable resource overhead. Furthermore, the limitation of deriving results solely from a single dataset [21] was evident. While they provided an AUC score, the absence of a direct recall metric was noticeable.

Cochrane et al. [30] employed DT, LR, and logistic regression to detect fraudulent activities. They leveraged the predicted values derived from each model, applying a unique formula that demonstrated superior outcomes in terms of both recall and precision, surpassing the performance achieved by each individual model. However, their study fell short in not reporting accuracy. Furthermore, their approach left potential room for further improvement in the recall metric.

In the present paper, we integrated KNN, LDA, and LR to enhance recall in comparison to other machine learning models by using conditional statements like “IF” and “THEN”, as well as operators such as “>“ and “<“. Our decision was influenced by the work of the authors [30], who developed an algorithm that utilized mean predicted values from LR in combination with DT and logistic regression models. This algorithm categorized rows as non-fraud if both DT and logistic regression models predicted “non-fraud” for a row and the LR predicted value for that row was lower than the mean of the LR predicted values across the entire dataset. Conversely, if both models predicted “fraud” for a row and the LR predicted value was higher than the mean, it was labeled as fraud. However, this approach had its limitations. Notably, the DT and logistic regression models required improved recall while still maintaining competitive accuracy levels with other machine learning models, necessitating adjustments to their algorithm to enhance performance. In this regard, achieving high recall is crucial in this context, where an imbalance between recall and accuracy can hinder generalization. Thus, our research aims to achieve a harmonious balance between high recall and accuracy.

## 3. Summary of the Proposed Strategy

### 3.1. Dataset Handling

We utilized four datasets from Kaggle, a prominent online community in the fields of machine learning and data science. Prior to feeding them into the algorithm, we partitioned all datasets into training data (80%) and test data (20%). To enhance the robustness of our methodology and maintain consistent model performance, we employed Stratified K-Fold cross-validation with a fold value of 5. Furthermore, we addressed the skewed nature of the credit card fraud-related data by dropping columns and filling missing values. It is important to note that this approach was chosen solely to demonstrate the performance superiority of our model.

#### 3.1.1. Synthetic Financial Datasets for Fraud Detection [18]

This dataset originated from Lopez-Rojas [18]. Given the scarcity of real-world financial datasets, he generated a synthetic dataset using the PaySim simulator. This dataset emulates typical transactions but incorporates certain malicious patterns. It is based on a sample of actual transactions extracted from a month’s worth of financial logs of a mobile financial service operating in an African country. We chose this dataset for our study due to its substantial data volume and because it has been employed in another study [30], allowing for direct model performance comparison. This dataset consists of 1,048,575 rows and 11 columns of data. Only the “type” column, representing event time, “nameOrig,” which anonymizes the customer initiating the transaction, and “nameDest”, which anonymizes the customer completing the transaction, were identified as categorical data. These three variables were transformed into numerical data using the LabelEncoder from the scikit-learn library. This covers the entirety of our preprocessing steps. The proportion of fraud cases within the dataset is 0.11%.

#### 3.1.2. Credit Card Transactions Fraud Detection Dataset [19]

**Figure 2:**
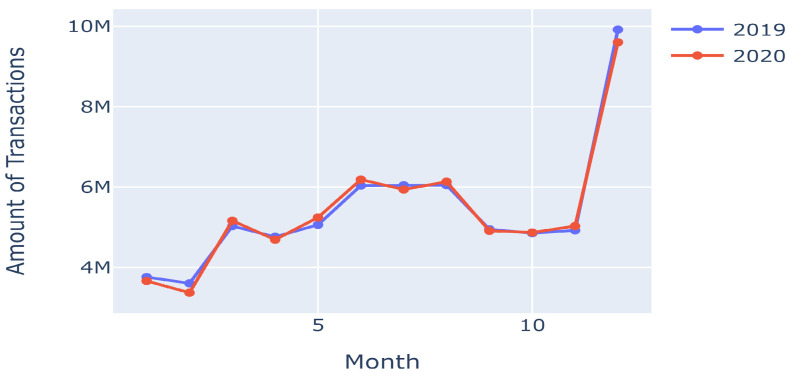
Time series analysis in the second dataset [19].

This dataset, introduced by Shenoy [19], was generated using the Sparkov Data Generation simulator. It encompasses 1,852,394 rows and 23 columns of data, making it suitable for time series analysis, as depicted in Figure 2. Several categorical variables (such as “merchant”, “category”, “first”, “last”, “gender”, “street”, “job”, “trans_num”, “city”, “state”, and “dob”) were converted into numerical data. Our preprocessing involved label encoding on these categorical variables, which covers all the preprocessing steps undertaken. The dataset contains a fraud case proportion of 0.52%.

#### 3.1.3. Credit-card-Fraud Detection Imbalanced Dataset [20]

**Figure 3:**
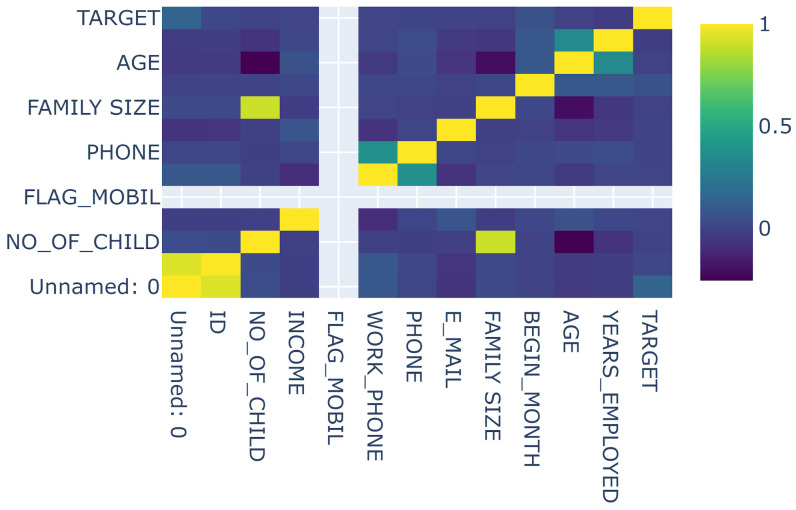
Visualization of the relationship between the columns in the third dataset [20].

Yadav [20] provides this dataset, containing 25,134 rows and 20 columns of data. The dataset features a fraud case proportion of 1.68%. It has several pertinent demographic variables, including family size, years employed, age, and number of children. Additionally, we have performed label encoding on columns such as “gender”, “car”, “reality”, “income_type”, “education_type”, “house_type”, and “family_type”, as these columns contain categorical variables. The relationship between the “TARGET” variable (the column holding predicted values) and the other columns is illustrated in Figure 3.

#### 3.1.4. IEEE_CIS Fraud Detection [21]

**Figure 4:**
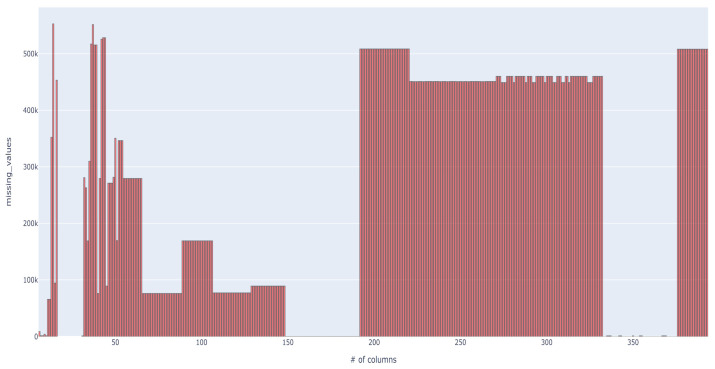
Missing values in the fourth dataset [21].

This dataset, provided by Vesta Corporation and the IEEE Computational Intelligence Society [21], is derived from Vesta’s real-world e-commerce transactions. It encompasses an extensive amount of data and variables, featuring both training and test subsets, yet our focus solely encompasses the training data because of the absence of fraud occurrence labels in the test data. With a total of 590,540 rows and 394 columns, this dataset contains numerous missing values, with 194 columns containing at least one such instance (Figure 4). We conducted a statistical analysis on the distribution of missing values across the dataset’s columns. Specifically, we found that the upper 25th percentile of columns held 460,110 missing values, the median had 168,969 missing values, and the lower 25th percentile contained 1269 missing values. Guided by these insights, we opted for a threshold grounded in the median: any column surpassing this median value of missing entries was excluded from our analysis. This approach was empirically validated to yield favorable performance outcomes. For the remaining columns, missing values were imputed using the median of the respective columns. The proportion of fraud cases in the dataset stands at 3.5%.

### 3.2. Description of the Models and Methodology

#### 3.2.1. Machine Learning Models

We employed KNN, LDA, and LR predictive models across all four datasets using the Python scikit-learn library. KNN is a straightforward yet powerful model that classifies or regresses new data points based on their proximity to the nearest neighbors in the training dataset. KNN can serve as an alternative to discriminate analysis when obtaining precise parametric estimates of probability densities is challenging [31]. The KNN process encompasses the following steps:

1. Collect training data.
2. Measure the similarity between the new input data and training data.
3. Choose the nearest K-neighbors.
4. Examine the labels of the selected nearest neighbors and classify or calculate the mean value for regression prediction.

In step 3, several methods can be utilized to select the nearest neighbors, including Euclidean distance, Manhattan distance, and cosine similarity. For instance, the Euclidean distance calculates the linear distance between two data points (Equation (2)):

(2) $$d\left(x,x^{\prime }\right)=\sqrt{{{(x}_{1}-{x^{\prime }}_{1})}^{2}+\ldots +{{(x}_{n}-{x^{\prime }}_{n})}^{2}}$$

This model requires careful tuning of the parameter K. If K is set too low, the risk of overfitting increases; conversely, if K is set too high, the classification performance might become inaccurate.

LDA is a linear classification model that employs supervised learning. It seeks to either maximize or minimize the scattering both between and within classes. The stages of LDA involve:

1. Calculate the scatter within classes and between classes. The within-class scatter matrix is defined by Equation (3), while the between-class scatter matrix is defined by Equation (4).
  (3) $$S_{W}={\sum_{i=1}^{C}\sum_{t=1}^{N}\left(x_{t}^{i}-\mu_{i}\right)(x_{t}^{i}-\mu_{i})}^{T}$$
  (4) $$S_{B}={\sum_{i=1}^{C}N(\mu_{i}-\mu )(\mu_{i}-\mu )}^{T}$$
2. Optimize the ratio of between-class variance to within-class variance by identifying vectors that maximize the separation between classes while minimizing the variance within each class.
3. Choose a new dimension and use the identified vectors to project data into a lower dimension, maximizing the separation between classes.
4. Identify the optimal vectors by computing the eigenvectors and eigenvalues of $S_{W}^{-1}S_{B}$, selecting those that maximize the separation between classes when data is projected onto them.

The LR model is outlined in Equation (5), where $h_{\theta }\left(x\right)$ denotes the predicted value, $\theta_{0}$, $\theta_{1}$, …, $\theta_{n}$ represent the weights, $x_{1}$, $x_{2}$, …, $x_{n}$ denote the features or attributes of the input data, and ε is the error term. In LR, the objective is to estimate the weights based on the provided dataset. This estimation predominantly employs the Ordinary Least Squares (OLS) method, aiming to ascertain weights that minimize the squared discrepancies between the actual values and the model’s predictions.

(5) $$h_{\theta }\left(x\right)=\theta_{0}+\theta_{1}x_{1}+\theta_{2}x_{2}+\ldots +\theta_{n}x_{n}+\varepsilon$$

#### 3.2.2. Our Proposed Methodology

The methodology involved in our algorithm entails the utilization of hyperparameters for machine learning models, selected based on their consistently strong performance across the datasets utilized. These settings are as follows. For KNN, we set the following hyperparameters: ‘algorithm’ as “auto”, ‘leaf size’ as “30”, ‘metric’ as “minkowski”, ‘metric_params’ as “None”, ‘n_jobs’ as “−1”, ‘n_neighbors’ as “5”, ‘p’ as “2”, and ‘weights’ as “uniform”. For LDA, the selected hyperparameters are as follows: ‘covariance_estimator’ as “None”, ‘n_components’ as “None”, ‘priors’ as “None”, ‘shrinkage’ as “None”, ‘solver’ as “svd”, ‘store_covariance’ as “False”, and the ‘tolerance’ as “0.0001”. For LR, we retained the default settings.


**Algorithm 1: Algorithm we made for better recall**
   **Input:**    pKNN = A predicted value from KNN     pLDA = A predicted value from LDA     pLR = A predicted value from LR     mvLR = A mean value from LR   **Output:**
   pOR = Predicted value from our methodology  FOR i FROM 0 to array of zeros with a length of a dataset DO      /*If “non-fraud” Comes Out from Both Models*/     IF (pKNN[i] is 0 OR pLDA[i] is 0) THEN       IF (pLR[i] < mvLR) THEN         pOR[i] ← 0       END IF     /*If “fraud” Comes Out from Both Models*/      ELSE IF (pKNN[i] is 1 OR pLDA[i] is 1) THEN       IF (pLR[i] > mvLR) THEN         pOR[i] ← 1        END IF      /*Allocating Predicted Values from KNN to Remainings*/      ELSE       pOR [i] ← pKNN[i]      END IF   END FOR

**Figure 5:**
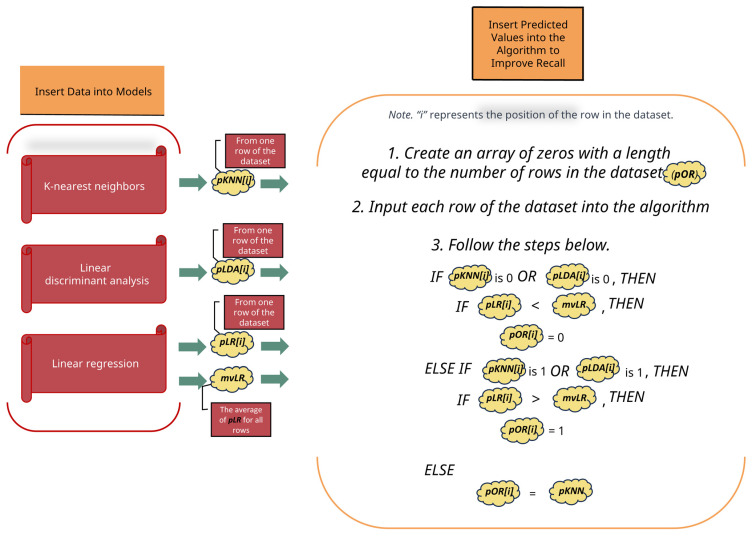
Additional description of Algorithm 1.

Algorithm 1, outlined above, presents the procedural steps employed in our methodology. To enhance understanding, Figure 5 is positioned above for more intuitive visualization. Our approach unfolds as follows: Initially, each dataset undergoes preprocessing. Upon introducing each dataset to the KNN, LDA, and LR models, a unique predicted value is assigned to every row of the dataset. We denote the predicted value from the KNN model as pKNN, from the LDA model as pLDA, and from the LR model as pLR. Given that both KNN and LDA are classifiers, their outputs can be anticipated to be discrete values like 0 or 1. On the contrary, LR, being a regression model, produces continuous values such as 0.1 or 0.6. Thus, pKNN and pLDA are expected to yield 0 or 1, while pLR will yield continuous values. Additionally, let us denote pKNN[i] as the predicted value obtained when the i-th row of the dataset is fed into the KNN model. For instance, pKNN[0] refers to the predicted value derived from the first row of the dataset, while pKNN[1] pertains to the predicted value derived from the second row.

Then, calculate the mean value (mvLR) of the predicted pLR values across all rows of the dataset. For instance, if a particular dataset has three rows with corresponding pLR values of 0.1, 0.3, and 0.2, then mvLR would be 0.2. Subsequently, we create an array called pOR, filled with zeros, having the same length as the number of rows in the dataset. For instance, if the dataset has five rows, pOR would be [0, 0, 0, 0, 0]. In this context, pOR[0] and pOR[1] would both be 0.

Now, we sequentially input each row of the dataset into our algorithm, processing the i-th row:

- If pKNN[i] is 0 or pLDA[i] is 0, and pLR[i] is less than mvLR, then pOR[i] is set to 0.
- Conversely, if pKNN[i] is 1 or pLDA[i] is 1, and pLR[i] is greater than mvLR, then set pOR[i] to 1.
- If neither of the conditions is met in a particular row, pOR[i] simply takes on the value of pKNN[i].
- As “i” progresses through the dataset rows, the pOR array is modified accordingly based on the logic applied.

Once all dataset rows have undergone this algorithm, the array, pOR, solidifies its values. This array could look like [0, 1, 1, ..., 0, 0, 1]. Now, by comparing the pOR values with predictions from other machine learning models on the dataset, performance metrics such as recall and accuracy can be evaluated.

## 4. Results and Setup

### 4.1. Results

For a comprehensive assessment of our model’s effectiveness, we conducted a rigorous comparison using the PyCaret against 61 traditional machine learning algorithms. This evaluation focused on key performance metrics, including recall, accuracy, and precision. Our configuration within the PyCaret environment involved specific settings: we set the “fold” value to ‘5’ and “session_id” to ‘0’. A “train_size” of ‘0.8’ was selected. To ensure experimental consistency with our methodology, we applied identical preprocessing to the dataset. The folding strategy employed was Stratified K-Fold. Additionally, as the data had already undergone preprocessing before being fed into the library, the “preprocess” feature was set to ‘False’. Our assessment ranked models based on their recall performance. We identified the top four models. Additional detailed information, including recall, accuracy, and precision for these models, is provided in Appendix A.

**Figure 6:**
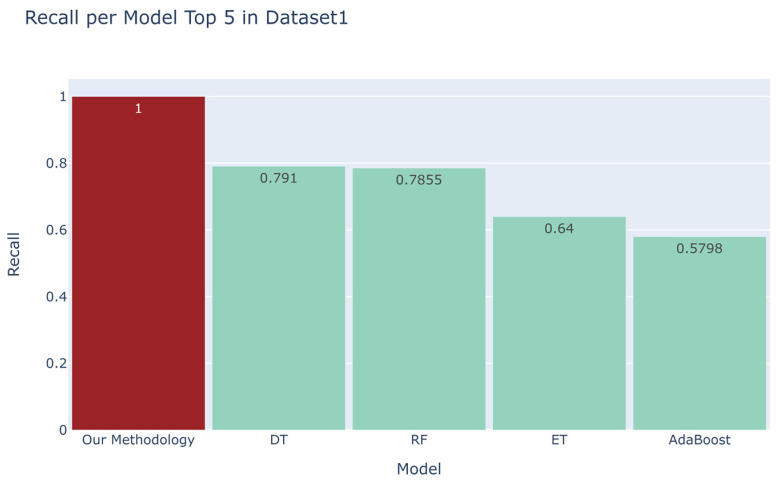
Comparison between the recall of our proposed methodology and that of the top four models in the automated machine learning library for the first dataset [18].

**Figure 7:**
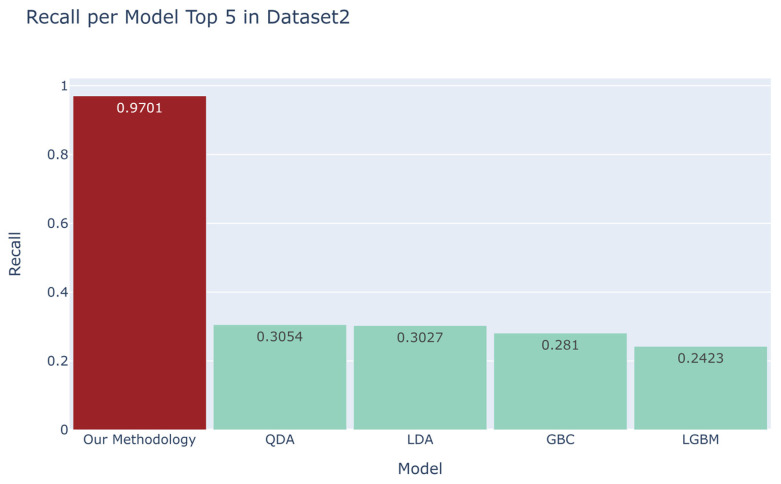
Comparison between the recall of our proposed methodology and that of the top four models in the automated machine learning library for the second dataset [19].

**Figure 8:**
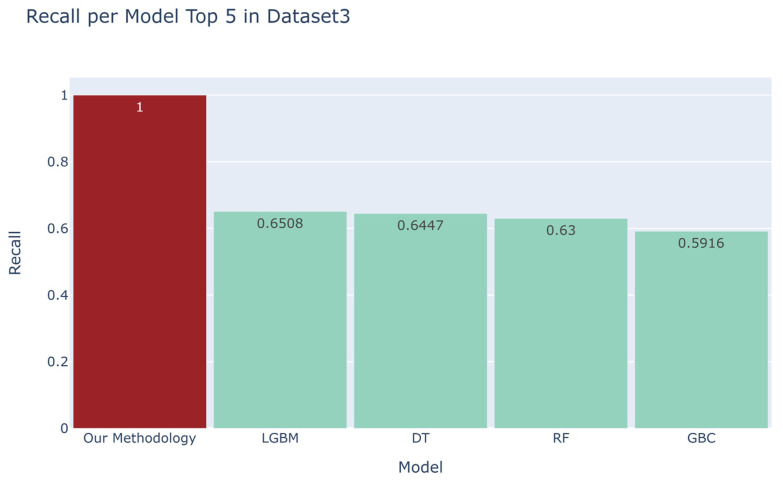
Comparison between the recall of our proposed methodology and that of the top four models in the automated machine learning library for the third dataset [20].

**Figure 9:**
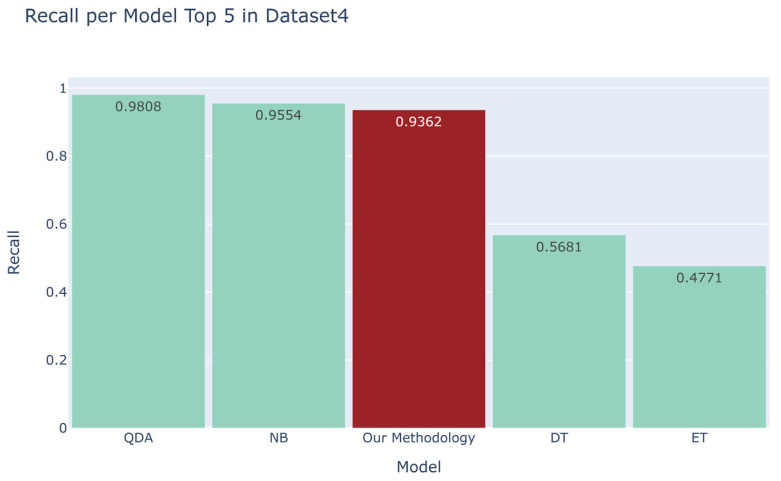
Comparison between the recall of our proposed methodology and that of the top four models in the automated machine learning library for the fourth dataset [21].

We compared the recall scores between the top four models derived from the automated machine learning library and our developed methodology (Figure 6, Figure 7, Figure 8 and Figure 9). For the first dataset, our approach attained a perfect score of 1.0000, while alternative models such as DT, RF, ET, and AdaBoost obtained scores of 0.791, 0.7855, 0.64, and 0.5798, respectively. Moving on to the second dataset, our methodology demonstrated a robust recall score of 0.9701, outpacing models such as Quadratic Discriminant Analysis (QDA), LDA, GBC, and LGBM, which yielded scores of 0.3054, 0.3027, 0.281, and 0.2423, respectively. Turning to the third dataset, our technique achieved a flawless recall score of 1.000, overshadowing the performance of models like LGBM, DT, RF, and GBC, which recorded scores of 0.6508, 0.6447, 0.63, and 0.5916, respectively. Lastly, in the fourth dataset, our methodology scored 0.9362, while competing models such as QDA, NB, DT, and ET garnered scores of 0.9808, 0.9554, 0.5681, and 0.4771, respectively. Additionally, as evidenced in Appendix A, our methodology exhibited commendable accuracy when benchmarked against other models across four distinct datasets [18,19,20,21], yielding accuracy scores of 0.9989, 0.9951, 0.9873, and 0.9664, respectively.

To sum up, our methodology outperformed other models in terms of recall in every dataset except for the fourth one [21]. In the fourth dataset, the NB and QDA models exhibited higher recall scores than our methodology. However, as discussed in Section 2.3, emphasizing high recall without a commensurate level of accuracy is not beneficial. As shown in Table 2, our methodology achieved an accuracy of 0.9656, which is significantly higher than that of 0.2783 from QDA and 0.0609 from NB. This clearly demonstrates the overall superiority of our methodology. Furthermore, the first dataset we employed had been previously utilized by the authors [30] referenced earlier. It is noteworthy that our perfect recall score of 1.0000 substantially exceeds their reported results, further underscoring the effectiveness of our approach.

### 4.2. Hardware and Software Setup

- Central Processing Unit: 13th Gen Intel^®^ Core™ i5-13500 2.50 GHz
- Random Access Memory: DDR5 32.0 GB
- JupyterLab 3.3.2
- Pandas 1.5.3
- Plotly 5.15.0
- PyCaret 3.0.4
- Python 3.9.7
- Scikit-learn 1.2.2

## 5. Discussion

Our innovative approach, which combines KNN, LDA, and LR, effectively enhances recall in credit card fraud detection without compromising accuracy. This method’s potential extends beyond credit card fraud detection, as its emphasis on achieving high recall can be valuable in other fields. Additionally, the versatility of our approach was demonstrated using tests across four distinct datasets.

However, our journey towards developing this solution was not without its setbacks. For instance, our initial attempts included incorporating a tree-based model into our algorithm, which performed well on some datasets but disappointingly on others. This led to the realization, as supported by previous research, that models like KNN and LDA hold the key to achieving strong recall performance, which contributed to our eventual success. Another challenge we faced involved modifying the conditional statements within our algorithm—specifically, the last condition that follows the initial ‘IF’ and subsequent ‘ELSE IF’ conditions. The recall score fluctuated significantly based on how this last condition was set. Initially, we explored a comparative approach between KNN and LDA for the final condition, only to find it counterproductive. In the end, a simple trial of assigning the pKNN[i] values to the remaining conditions yielded surprisingly positive results. This experience reinforced our belief that sometimes a straightforward approach, even with simple models like KNN and LDA, can produce effective results.

Despite its strengths, our study does have a limitation concerning precision. Given the well-established trade-off between recall and precision [32], the relationship between these metrics is reflected in the data in Appendix A. Future research is thus needed to develop strategies that focus on enhancing precision for specific objectives, thereby offering a promising avenue for further exploration. Additionally, there are a few limitations that warrant discussion. First, while we compared our methodology with models from an automated machine learning library, it is unclear whether our approach aligns or benchmarks against state-of-the-art models explicitly designed for fraud detection. Second, it is unfortunate that we did not employ techniques such as regularization, oversampling, and undersampling methods in this experiment to address skewed datasets.

## 6. Conclusions

This study proposed a methodology aimed at enhancing recall in credit card fraud detection across four distinct datasets. By preprocessing these datasets and prioritizing high recall while maintaining accuracy, our model yielded recall scores of 1.0000, 0.9701, 1.0000, and 0.9362 for the respective datasets. Our approach demonstrated competitive accuracy compared to other models. The availability of the datasets we utilized on the platform Kaggle holds the potential for guiding future fraud detection strategies. We hope to see our method applied in various fields where recall is essential, such as medical diagnostics, disaster forecasting, and airport security.

Looking ahead, we anticipate vast opportunities to extend our method. We envision its integration into a dynamic and adaptable framework, enabling real-time fraud detection with applications in online banking and other domains. The intrinsic versatility of our methodology suggests potential applicability across diverse areas, including internet banking, e-commerce platforms, and the rapidly evolving mobile payment systems.

## Figures and Tables

**Table 1:** Comparison of the related studies addressing credit card fraud datasets.

Author	Year	Method	Shortfall
Zahoora et al. [23]	2022	Achieved a high recall value in detecting zero-day ransomware by employing a self-made voting mechanism. This mechanism was a combination of Contractive Autoencoder (CAE) and four combination rules.	Due to the limited scope of the dataset they utilized, the generalizability of their method cannot be assured when applied to entirely different datasets.
Verma and Chandra [24]	2023	Proposed a RepuTE Framework aimed at enhancing trust in fog computing, using a soft-voting ensemble model to classify and predict DoS/DDoS and Sybil attacks. The model achieved a 99.99% accuracy rate, outperforming existing solutions.	In imbalanced scenarios like credit card fraud datasets, the method’s effectiveness is uncertain.
Malik et al. [25]	2022	Evaluated performance from seven hybrid models, which are in conjunction with AdaBoost, and found that AdaBoost combined with LGBM shows high performance in terms of ROC score.	No mention of accuracy. As will be elaborated later in this study, while the NB model shows high recall, its accuracy is conspicuously low.
Jiang et al. [26]	2023	Proposed a novel unsupervised attentional anomaly detection network-based framework for credit card fraud detection (UAAD-FDNet) designed to achieve high precision, recall, F1 score, and AUC.	Recall is relatively low compared to precision, F1 score, and AUC score. It could have been higher. One dataset was employed.
Akshaya et al. [27]	2022	Comparing the predictive performance of various models, including logistic regression, GBC, KNN, and RF, it was found that a voting classifier, which leverages these aforementioned models, yields the highest accuracy and F1 score.	In terms of recall, the voting classifier presented low performance according to their data.
Cai and He [28]	2022	Proposed a hybrid model in conjunction with XGBoost and TabNet after replacing the missing value with −999 in order to reach a high AUC score and accuracy.	Comparison of performance was implemented with only four models and one dataset. AUC score and accuracy were only considered, and detailed recall was not introduced.
Nguyen et al. [29]	2022	Evaluated the AUC score using catboost and deep neural networks after categorizing credit card users as either old or new. Feature engineering and transformations were also implemented to achieve a high AUC score.	Detailed information about recall and precision was not provided, and only one dataset was employed.
Cochrane et al. [30]	2021	Combined predicted values from LR, DT, and logistic regression models and applied a particular formula to elevate recall and precision.	Only recall and precision are considered, and accuracy is not mentioned. The performance could have been higher.

**Table 2:** Comparison of the accuracy and recall between our methodology, QDA, and NB.

Index	Model	Accuracy	Recall
1	Our Method	0.9664	0.9362
2	QDA	0.1135	0.9808
3	NB	0.0500	0.9554

## Abbreviations

The following abbreviations are used in this manuscript:

DT	Decision Tree
ET	Extra Trees
GBC	Gradient Boosting Classifier
KNN	K-Nearest Neighbor
LDA	Linear Discriminant Analysis
LGBM	Light Gradient Boosting Machine
LR	Linear Regression
NB	Naive Bayes
QDA	Quadratic Discriminant Analysis
RF	Random Forest
SVM	Support Vector Machine

## Appendix A

**Table A1:** Recall results for our proposed method and the top four models from the automated machine learning library PyCaret.

Index	Dataset #	Top #	Model	Recall	Accuracy	Precision
1	1	1	Our Method	1.0	0.9989	0.0656
2	1	2	DT	0.7910	0.9996	0.8036
3	1	3	RF	0.7855	0.9998	0.9853
4	1	4	ET	0.6400	0.9996	0.9982
5	1	5	AdaBoost	0.5798	0.9995	0.9549
6	2	1	Our Method	0.9701	0.9951	0.0635
7	2	2	QDA	0.3054	0.9900	0.1938
8	2	3	LDA	0.3027	0.9907	0.2092
9	2	4	GBC	0.2810	0.9956	0.6450
10	2	5	LGBM	0.2423	0.9949	0.4906
11	3	1	Our Method	1.0	0.9873	0.2440
12	3	2	LGBM	0.6508	0.9931	0.9149
13	3	3	DT	0.6447	0.9861	0.5822
14	3	4	RF	0.6300	0.9926	0.9052
15	3	5	GBC	0.5916	0.9925	0.9476
16	4	1	QDA	0.9808	0.1135	0.0373
17	4	2	NB	0.9554	0.0500	0.0340
18	4	3	Our Method	0.9362	0.9664	0.0429
19	4	4	DT	0.5681	0.9666	0.5207
20	4	5	ET	0.4771	0.9801	0.9137
